# Which Vitamin D in CKD-MBD? The Time of Burning Questions

**DOI:** 10.1155/2013/864012

**Published:** 2013-08-07

**Authors:** Andrea Galassi, Antonio Bellasi, Sara Auricchio, Sergio Papagni, Mario Cozzolino

**Affiliations:** ^1^Medical Department, Nephrology Unit, AO Desio Vimercate, Desio Hospital, 20832 Desio, Italy; ^2^Department of Nephrology, Sant'Anna Hospital, 22020 Como, Italy; ^3^Department of Health Sciences, University of Milan, 20142 Milan, Italy; ^4^Division of Nephrology, Dialysis Center CBH-Città di Bisceglie, 70052 Bisceglie, Italy

## Abstract

Vitamin D is a common treatment against secondary hyperparathyroidism in renal patients. However, the rationale for the prescription of vitamin D sterols in chronic kidney disease (CKD) is rapidly increasing due to the coexistence of growing expectancies close to unsatisfactory evidences, such as (1) the lack of randomized controlled trials (RCTs) proving the superiority of any vitamin D sterol against placebo on patients centered outcomes, (2) the scanty clinical data on head to head comparisons between the multiple vitamin D sterols currently available, (3) the absence of RCTs confirming the crescent expectations on nutritional vitamin D pleiotropic effects even in CKD patients, (4) the promising effects of vitamin D receptors activators (VDRA) against proteinuria and myocardial hypertrophy in diabetic CKD cohorts, and (5) the conflicting data on the impact on mortality of VDRA versus calcimimetic centered regimens to control CKD-MBD. The present review arguments these issues focusing on the opened questions that nephrologists should consider dealing with the prescription of nutritional vitamin D or VDRA and with the choice of a VDRA versus a calcimimetic based regimen in CKD-MBD patients.

## 1. Introduction

Secondary hyperparathyroidism (SHPT) is recognized as a major complication of chronic kidney disease (CKD). Over the past decades, Nephrologists have been encouraged to effectively control PTH due to the reported worrisome consequences of SHPT as brown tumors, severe cardiac hypertrophy, bone pain, skeletal fractures, and calciphylaxis. Although repeated observational data described an independent association between PTH levels and unfavorable outcomes in CKD stage 3–5 [1–3] as well as in ESRD patients [4, 5], no randomized controlled trials (RCTs) have still proven that an active reduction of PTH values could improve patient-centered outcomes as hospitalizations, cardiovascular events (CVE), CKD progression, and survival. Furthermore, the optimal targets of PTH levels are still uncertain in CKD as well as in ESRD cohorts. Thus, KDIGO guidelines provide a low-grade suggestion to maintain PTH levels into the range of normality in CKD 3–5 and between 2 and 9 times the normal range in ESRD [6]. Active vitamin D receptor activators (VDRA) are one of the classic therapies suggested to achieve those PTH targets [6]. 

**Figure 1:**
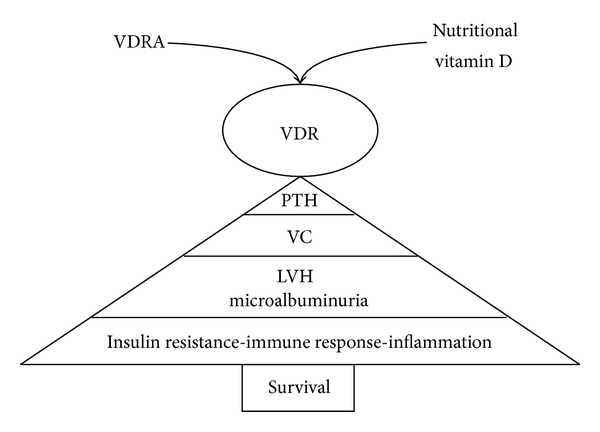
The growing targets of active and native vitamin D. LVH: left ventricular hypertrophy; VC: vascular calcification; VDR: vitamin D receptor; VDRA: vitamin D receptor activators.

Emerging evidence of several pleiotropic effects related to the activation of the vitamin D receptor (VDR) is transforming the original world of vitamin D into a more complex scenario and affecting the use of vitamin D sterols among nephrologists. Different forms of vitamin D analogs are currently available in several countries, but clinical data on head to head comparisons between them are still scanty. Nonetheless, promising data suggest some beneficial effects of vitamin D analogs on proteinuria, myocardial hypertrophy in diabetic CKD cohorts, inflammation, and cardiorenal syndromes (Figure 1) [7, 8]. Nutritional vitamin D replenishment is also receiving a growing interest for its potential autocrine-paracrine effects even in CKD patients, although its use is still based on observational rather than RCT data. Finally, the advent of calcimimetics opened the crucial debate on the potential benefits offered by a VDRA in respect of a calcimimetic based regimen against CKD-MBD and mortality in dialysis patients.

The present review will argue these issues, focusing on the open questions that nephrologists should consider dealing with the prescription of nutritional vitamin D or VDRA and with the selection of a VDRA versus a calcimimetic based regimen in CKD-MBD patients.

## 2. Vitamin D Metabolism in Humans and Vitamin D Sterols Currently Available in Nephrology Area

Humans derive vitamin D from exposure to sun light and, into a lesser extent, from the diet. The term “nutritional vitamin D” refers to 25(OH)D_2_ and 25(OH)D_3,_ the natural precursors of their active forms, 1,25(OH)_2_D_2_ and 1,25(OH)_2_D_3_, respectively, which are thereafter capable to activate the VDR. 25(OH)D_2_ and 25(OH)D_3_ are specifically transformed into 1,25(OH)_2_D_2_ and 1,25(OH)_2_D_3_ respectively by the renal and, into a lesser extent, by the extrarenal 1 alpha hydroxylase [9]. 25(OH)D_2_ and 25(OH)D_3_ derive from the hepatic hydroxylation in the 25th position of their two precursors, ergocalciferol, and cholecalciferol, respectively [9]. Ergocalciferol derives from the UV irradiation of the yeast sterol ergosterol, naturally found in sun-exposed mushrooms, while cholecalciferol from UVB irradiation of the 7-dehydrocholesterol [10]. Notably, humans do not synthesize vitamin D2 [10]. Almost 80% of vitamin D is obtained by UVB irradiation with only a minor contribution of diet intake [11].

 Several vitamin D sterols are currently available for prescription in a variety of medical areas (Table 1). Ergocalciferol, cholecalciferol, and calcifediol (nutritional vitamin D) can be currently prescribed to replenish lower levels of circulating 25(OH)D_3_. Nutritional forms of vitamin D are nowadays flanked by several VDRA, which can activate VDR directly or after a previous hydroxylation step. Calcitriol is the natural VDRA [1,25(OH)_2_D_3_], capable to activate the VDR without any prior hydroxylation process, historically adopted to control SHPT in renal patients. More recently, four vitamin D analogs have been introduced in the nephrology arena, namely, alfacalcidol [1*α*(OH)D_3_], doxercalciferol [1*α*(OH)D_2_] paricalcitol [19-nor1,25(OH)_2_D_3_], and oxacalcitriol [22oxa1,25(OH)_2_D_2_]. Alfacalcidol [1*α*(OH)D_3_] and doxercalciferol [1*α*(OH)D_2_] require to be hydroxylated in 25 position prior to activate the VDR, while paricalcitol [19-nor1,25(OH)_2_D_3_] and oxacalcitriol [22oxa1,25(OH)_2_D_2_], derived from the side chain modification of 1,25(OH)_2_D_2_ and 1,25(OH)_2_D_3_, respectively, elicit a peculiar activation of the VDR expressed at the parathyroid gland with a lesser effect on those expressed in the intestinal tract. 

Each of these vitamin D sterols deserves specific characteristics, which should be taken into account by nephrologists while approaching any vitamin D regimen.

## 3. Nutritional Vitamin D: Protective Replenishment or Active Therapy?

 Nutritional vitamin D has received an exponential interest of scientific press during the last ten years. The medical literature yearly hosts tenths of papers describing associations between circulating levels of 25(OH)D and a variety of diseases, from osteoporosis [12] to hypertension [13], cardiovascular disease [14], insulin resistance [15], infections [16], cancer [17] and mortality [18]. 25(OH)D deficiency has been similarly linked to CKD progression [19], SHPT [20], and survival [21] in renal patients. The widespread association between 25(OH)D and unfavorable outcomes in the general population, as well as in selected diseased subcohorts, inspired a potential role of nutritional vitamin D as an etiologic factor rather than a mere biomarker of clinical debacles. The emerging knowledge about the extrarenal activation of 25(OH)D, prompting the calcitriol-related genomic effects at an autocrine and paracrine level, extended the hypothesis of such a causal link also in CKD cohorts [22]. Nutritional vitamin D (cholecalciferol, ergocalciferol, or calcifediol) is nowadays prescribed when 25(OH)D levels fall under 30 ng/mL in the community as well as in CKD and ESRD patients. However, the desirable targets of 25(OH)D are still prone to current revaluations [12, 23, 24]. KDIGO guidelines provide a *not graded* suggestion to replenish 25(OH)D deficiency as the first step to correct SHPT in CKD stage 3–5 [6], while no suggestions are provided on the administration of nutritional vitamin D in dialysis patients. Although these relevant associations, crucial aspects still require attention prior to trace enthusiastic conclusions about the effect of nutritional vitamin D replenishment on patient-centered outcomes in CKD-ESRD patients. 

Levels of 25(OH)D > 30 ng/mL are considered sufficient, levels of 21 to 29 ng/are defined as insufficient, while levels below 20 ng/mL are considered deficient [9]. 25(OH)D deficiency is highly prevalent in the general population as well as in CKD, exceeding 80% in dialysis patients [25]. Physicians can actually replenish 25(OH)D deficiency with the 3 nutritional drugs: ergocalciferol, cholecalciferol, and calcifediol. Several studies observed that ergocalciferol was less potent than cholecalciferol in achieving 25(OH)D targets [26]. This could be due to a stronger affinity of cholecalciferol to the vitamin D binding protein [27]. Furthermore, the activation of vitamin D receptor (VDR) by 1-25(OH)_2_D_3_ could be more sustained due to the capability of 1-24-25(OH)_3_D_3_ to still activate VDR [27], contrarily to what is observed for 1-24-25(OH)_3_D_2_ [28]. Thus, 50.000 IU of ergocalciferol are purposed as equivalent to 15.000–5.000 IU of cholecalciferol [29]. Notably, in a recent post hoc analysis by Biancuzzo et al. of a previously published double-blind placebo-controlled trial, ergocalciferol 1000 UI/day for 11 weeks was equivalent to cholecalciferol at the same dose in improving the circulating levels of 25(OH)D [30]. Two registered RCTs are actually recruiting patients to compare the effect of vitamin D2 against vitamin D3 on mineral metabolism in CKD stage 2–5 (NCT01633853, NCT01173848). Notably, the choice of the “prodrugs” ergocalciferol or cholecalciferol, compared to calcifediol, could be more protective against the risk of further calcitriol intoxication. Particular attention is always mandatory while prescribing nutritional vitamin D in the cases of suspected 1-alpha-hydroxylase overexpression, as it is for sarcoidosis or B-cell lymphomas. Nigwekar et al. have recently proposed an 8 to 16 weeks regimen to replenish 25(OH)D deficiency in CKD patients, starting with 50.000 IU ergocalciferol or 10.000 IU cholecalciferol weekly [29].

 Nutritional Vitamin D can be activated also in renal patients by the extrarenal 1-alpha-hydroxylase, mainly expressed in monocytes/macrophages and parathyroid cells [22]. According to the recent meta-analysis by Kandula and colleagues, observational studies demonstrate that nutritional vitamin D reduces PTH levels in CKD (−24.24 pg/mL) as well as in ESRD (−59.49 pg/mL) patients [31]. However, the studies were heterogeneous for the nutritional vitamin D prescription in terms of type, doses, and route of administration [31]. More recently Alvarez and colleagues randomized 48 CKD 2-3 patients to cholecalciferol versus placebo [32]. Cholecalciferol improved SHPT compared to placebo up to one year of followup [32]. The entity of PTH reduction suggests a significant impact of nutritional vitamin D administration only for mild SHPT, being limited to an adjuvant role in the majority of other cases. The early replenishment of nutritional vitamin D deficiency could rather be preventive, delaying the further onset and evolution of SHPT [33]. Observational data from Tassin showed a progressive reduction of PTH levels in incident dialysis patients since 2004 to 2009, parallel to the increased prevalence of cholecalciferol and calcifediol prescription in the same incident individuals [34]. 

VDRA are expected to elicit a stronger PTH reduction compared to their native forms. The Cochrane meta-analysis by Palmer et al. reported a 196.05 pg/mL reduction of PTH levels in dialysis patients, receiving VDRA [35], which was three times higher than that reported for nutritional vitamin D [31]. Paricalcitol and doxercalciferol induced a stronger PTH reduction compared to ergocalciferol and cholecalciferol in CKD 3-4 patients respectively [36, 37]. However, the comparison between nutritional vitamin D and VDRAs in achieving the suggested PTH targets needs to be deepened in further RCTs. 

Data concerning PTH reduction by the coadministration of nutritional vitamin D and VDRA are still heterogeneous and mainly acquired in observational fashion [38]. Furthermore, only a subset of patients concomitantly received the nutritional and the active vitamin D in those studies [39]. Calcifediol was associated with a significant PTH reduction and a lowering rate of alfacalcidol administration from 66% to 43% in 149 dialysis patients during 6 months of followup [40]. Contrarily, cholecalciferol 20.000 IU/week did not impact on PTH reduction in 64 dialysis patients, 40 of whom were undergoing calcitriol or alfacalcidol [41]. PTH remained similarly unchanged in 42 dialysis patients on VDRA randomized to nutritional vitamin D (10.333 IU cholecalciferol/week) versus placebo [42]. Matias et al. investigated 158 dialysis patients (44% receiving paricalcitol) treated with one year cholecalciferol administration at doses tailored to basal 25(OH)D levels, with a maximum of 50.000 IU per week [43]. A significant mild decrease in iPTH levels (from 233 to 208 pg/mL, *P* < 0.001) was accompanied by a mild reduction in the doses of paricalcitol [43]. Finally, a short run with very high doses of cholecalciferol (200.000/week for 3 weeks) resulted similar to placebo in terms of PTH reduction at 6 weeks despite the increase of 25(OH)D up to 52.4 ± 18 ng/mL [44]. Of note, some studies observed a mild, but significant, increase of calcium levels during nutritional vitamin D administration [40, 41, 45]. More recently, Delanaye et al. observed a significant difference in the 12 months PTH reduction among 43 hemodialysis patients randomized to cholecalciferol 25.000 UI every two weeks [−115 pg/mL (95% CI: −192 to 81)] versus placebo [+80 pg/mL (95% CI: −58 to 153)] (*P* = 0.02) [46].

The effect of nutritional vitamin D on bone histology of renal patients has been poorly investigated. Coen et al. retrospectively observed a reduction in bone turnover of dialysis patients with 25(OH)D levels higher than 40 ng/mL [47]. Specific RCTs are advocated to test this relevant issue.

VDRAs consistently increase FGF23 levels [48–50] in predialysis patients. Nutritional vitamin D seems to elicit a similar effect, which could be however different according to the type and the regimen of vitamin D adopted [32, 51]. The clinical relevance of FGF23 variations induced by vitamin D is an intricate topic beyond the purposes of this review.

Great expectations are placed in the possible pleiotropic effects of nutritional vitamin D. The synthesis of extrarenal 1-alpha hydroxylase in multiple cell lines and the ubiquitous expression of VDR suggest that the 25(OH)D provision could sustain its activation, favoring the VDR-mediated genomic effect of calcitriol at an autocrine and paracrine level [22]. Kim et al. observed a 25% reduction of urinary albumin-to-creatinine ratio (UACR) in CKD diabetic patients randomized to cholecalciferol against placebo on top of ACE-I or ARBs [52]. Cholecalciferol administered at high doses was heterogeneously associated with improved left ventricular hypertrophy [45], erythropoietin doses [43], endotoxin activity [53], and inflammation [45, 54]. Several RCTs are currently investigating the potential effect of nutritional vitamin D on LVH (NCT01323712), insulin resistance (NCT00893451), erythropoietin dosing (NCT01395823), proteinuria (NCT01426724) immunity (NCT00892099), arterovenous fistulae maturation (NCT00912782), and physical and cognitive performance (NCT00511225, NCT01229878). 

A recent meta-analysis described a 14% increased mortality risk for each 10 ng/mL reduction of 25(OH)D levels in CKD cohorts [21]. Although vitamin D replenishment is generally adopted at levels inferior to 30 ng/mL in renal patients, certain studies showed an increased risk of ESRD [19] and mortality [55] at lower 25(OH)D levels. Though the association between 25(OH)D levels and mortality was attenuated in dialysis patients receiving VDRA [55], no RCTs have investigated the effect of nutritional vitamin D supplementation on survival in renal ESRD cohorts. The NUTRIVITA study is randomizing Italian dialysis patients to calcifediol versus control to test the effect of treatment on survival, nonfatal myocardial infarction, and stroke (NCT01457001).

While awaiting for new evidences, the not graded KDIGO suggestion to supplement nutritional vitamin D to achieve levels of 25(OH)D of at least 30 ng/mL seems acceptable as a complementary step in SHPT management, especially in the early stages of CKD. However, the following burning questions still deserve to be answered by dedicated RCTs (Table 2):

1. What are the optimal levels of 25(OH)D to aim for to ameliorate both SHPT control and survival in renal patients?
2. What is the best nutritional vitamin D type (cholecalciferol, ergocalciferol, or calcifediol) and dose regimen to correct 25(OH)D deficiency and treat SHPT?
3. Is the correction of 25(OH)D deficiency in the earlier stages of CKD cost-effective to prevent CKD-MBD?
4. Does nutritional vitamin D supplementation improve CKD progression, diabetes, infections, and survival in renal patients?
5. Is the coadministration of nutritional vitamin D and VDRA associated with additive benefits on CKD-MBD, infections, and diabetes control as well as mortality in CKD patients?

## 4. VDRA: A Multifaceted Choice from Secondary Hyperparathyroidism to Survival

 The reduction of serum 1,25(OH)_2_D and calcium levels together with total body phosphate expansion are the major causes of SHPT. Hence, VDRA are traditionally considered a cardinal treatment of SHPT. KDIGO guidelines suggest starting a VDRA in case of raising PTH values in the course of CKD [6]. On the contrary, its suspension or reduction is suggested in the case of hypercalcemia or hyperphosphatemia [6]. The risks related to high doses of vitamin D are mainly due to phosphate and calcium overload which are both associated with worse survival in dialysis patients [56, 57]. Furthermore, the achievement of the suggested calcium and phosphate targets is still suboptimal in the European dialysis population, as reported by the recent COSMOS investigation [58]. Thus, selective VDRA with a stronger effect on PTH and a lesser impact on calcium and phosphate load may improve the global achievement of mineral targets reducing the toxicity of high vitamin D dosage.

In the recent years, industries have provided multiple synthetic vitamin D, namely, vitamin D2 (paricalcitol and doxercalciferol) and vitamin D3 analogs (alfacalcidol, falecalcitriol, and oxacalcitriol) (Table 1). These molecules actually received a growing interest for their capability to control PTH values with a lower impact on phosphate and calcium levels compared to calcitriol [59–61] in some instances reported similar to placebo [62–64]. However, many questions are still opened about the comparison between different vitamin D analogs on mineral metabolism, surrogate endpoints, and patient-centered outcomes.

Although an encouraging superiority of single analogs against placebo and calcitriol in controlling PTH, calcium, and phosphate, only few studies compared the impact of different analogs on serum CKD-MBD targets, leading to heterogeneous and inconclusive results. Alfacalcidol was similar to calcitriol in suppressing PTH values with equal change in phosphate and calcium levels [65, 66], but recent data by Hansen et al. did not observe any significant difference between alfacalcidol and paricalcitol on similar targets [67]. Joist et al. observed that paricalcitol at very high doses, far from those commonly adopted in clinical practice, suppressed PTH with lower elevation of phosphate and calcium levels compared to doxercalciferol [68], but the conversion from intravenous paricalcitol to doxercalciferol resulted in equally satisfactory control of PTH, calcium and phosphate [69]. No clinical data comparing doxercalciferol with alfacalcidol in humans are currently available. 

Different vitamin D analogs may induce a peculiar activation of VDR with substantial different effects on the vascular calcification (VC) and bone remodeling pathways. Experimental data observed that VC and arterial stiffness were less pronounced in rats treated with paricalcitol, compared to those receiving other VDRAs [70–72]. Paricalcitol resulted also bone protective in experimental models compared to vehicle [73] and with a lower risk of adynamic bone disease compared to calcitriol [74], doxercalciferol [75], or cinacalcet [38]. However, the expected effect of different vitamin D analogs on bone and vascular health are still unexplored in humans.

More recently nutritional vitamin D and VDRA received growing interest for their potential pleiotropic effects, related to the widespread regulation of human genome played by VDR activation. Albuminuria and left ventricular hypertrophy (LVH) of diabetic CKD patients emerged as new targets of vitamin D analogs [76]. The activation of VDR can actually regulate the expression of several genes involved in glomerular and myocardial inflammation as renin [77], TGF-beta [78], antioxidant molecules [79], NF*κ*B, and RANTES [80]. The VITAL study, a randomized placebo controlled trial in diabetic CKD patients, confirmed a significant, dose dependent and reversible reduction of albuminuria when paricalcitol was added to RAAS inhibitors [81]. More recently a post hoc analysis of the PRIMO study observed a lower increase of brain natriuretic peptide and left atrial index in diabetic CKD patients receiving paricalcitol on top of ACE-I or ARBs compared to placebo [82]. Interestingly, paricalcitol was associated with lower risk of hospitalization in those patients with more severe LVH [83]. Of note, paricalcitol resulted associated with significant reduction of oxidative stress [84] and improved peritoneal membrane permeability in hemodialysis and peritoneal dialysis, respectively [85]. However, no RCTs have tested the effect of paricalcitol against other forms of vitamin D on albuminuria and LVH. Furthermore, no RCTs have investigated if the benefits offered by paricalcitol against placebo on albuminuria and LVH may improve hard patient-centered outcomes.

The aforementioned data suggested potential benefits offered by vitamin D analogs on hospitalization, cardiovascular events, and mortality. Several observational data support these hypotheses. A 14% reduction in all-cause hospitalization was observed in hemodialysis patients receiving paricalcitol compared to those treated with calcitriol [5]. Paricalcitol [86–88] and doxercalciferol [86] resulted associated with lower mortality compared to calcitriol. However, in the DOPPS cohort VDRA administration was not associated with improved survival in models more independent of unmeasured confounders as comorbidities [89]. Results from the Italian FARO survey unexpectedly showed a better survival in dialysis patients receiving VDRA also in the presence of PTH ≤ 150 pg/mL [90]. The evidence-based approach requires further RCTs to confirm these observational data prior to orient stronger recommendations on VDRA prescription. However, while the head to head comparison between different VDRA would be ethically acceptable, the direct comparison between VDRA versus placebo in the absence of other treatments against SHPT as calcimimetics would be ethically questionable for the reason mentioned previously. This ethical conundrum still entraps the birth of new RCTs to support higher grade recommendations on vitamin D prescriptions. 

The adoption of VDRA appears heterogeneous across Europe. Recent data from the COSMOS study presented a limited use of VDRA in European dialysis patients (48%), with calcitriol and alfacalcidol accounting for 93.3% and paricalcitol for 6.7% [58]. The adoption of alfacalcidol was double that of calcitriol in the non-Mediterranean countries, and the opposite was seen in Mediterranean areas [58]. Data form the Italian FARO survey describe an increase in the prescription of iv paricalcitol from 24.4% to 41.9% and a consensual reduction in the prescription of calcitriol since April 2006 to October 2007, among dialysis patients with iPTH > 150 pg/mL [90]. Similar trends are reported by the United Renal Data System between 1999 and 2008, with a declined use of intravenous calcitriol from 83.9% to 1.8%, accompanied by a raise in paricalcitol and doxercalciferol intravenous adoption [91]. Health-economic analysis observed that paricalcitol may have potential economic benefits in both CKD and dialysis patients compared to calcitriol and alfacalcidol [92, 93]. Furthermore, a recent economic analysis of the FARO survey observed that intravenous paricalcitol was more cost-effetctive compared to the combination of paricalcitol plus cinacalcet [94]. However, any economic consideration is far from being conclusive, coming from observational data rather than from controlled intervention. 

 In the authors opinion, this body of insights raises the following questions, which still deserve to be investigated in further RCTs (Table 2):

1. Is any VDRA superior to placebo in terms of cardiovascular events and survival?
2. Do vitamin analogs provide a better achievement of patient centered outcomes compared to calcitriol?
3. Is any vitamin D analog superior to the others in achieving KDIGO targets and improving albuminuria, LVH, VC, CVE, bone health, hospitalizations, and survival?
4. Will paricalcitol ameliorate CKD progression and cardiovascular events through the benefits on albuminuria and LVH?
5. Should VDRA be suspended in those patients reaching PTH levels ≤ 150 pg/mL?

## 5. VDRA and/or Calcimimetic: The Open Debate

Differences between a VDRA versus a calcimimetic-centered regimen represent an open debate in the management CKD-MBD in dialysis patients [95]. The main terms of this comparison are focused on SHPT control, progression of VC, and survival.

Two recent RCTs, the ACHIEVE [96] and the IMPACT [97] study, investigated the effect of these regimens against SHPT, leading to conflicting results. The SHPT control resulted superior in the cinacalcet and in the paricalcitol iv centered group in the ACHIEVE and the IMPACT trial respectively. Notably, some major differences in the two study designs may account for these discrepant results: (1) in the ACHIEVE study the active VDRA group included both paricalcitol and doxercalciferol, while paricalcitol was the only VDRA accepted in the D arm of the IMPACT study, (2) cinacalcet was admitted for hypercalcemia during VDRA in the IMPACT study, while it was excluded from the D arm in the ACHIEVE study, and (3) the laboratory parameter thresholds selected to introduce or suspend cincalacet and VDRA were different in the two trials. On the opposite, both the studies presented higher rates of hypocalcemia and increasing adoption of calcium-based phosphate binders in the cincalacet arm. In light of the study design differences, these results do not allow absolute conclusions about the potential superiority of any of these two approaches against SHPT. 

The recently published ADVANCE trial [98] investigated whether cinacalcet in combination with low dose of VDRA (<6 mcg paricalcitol equivalent/week) versus flexible doses of VDRA attenuates coronary, aorta, and cardiac valves calcification progression in 360 hemodialysis patients. After 12 months of followup, a trend toward CAC reduction in the cinacalcet arm was noted [24% (95% CI: −22% to 119%)] and [31% (95% CI: −9% to 179%)], in the cinacalcet and flexible vitamin D group, respectively (*P* = 0.073). Of interest, as in the ACHIEVE study, in the ADVANCE study protocol no specific recommendation on vitamin D administration was made resulting in an heterogeneous use of different forms of VDRA. Finally, the large dose of calcium containing phosphate binders and vitamin D administered in the calcimimetic arm further complicate the interpretation of these results [99]. 

The effect on hard endpoints offered by cinacalcet versus placebo, on top of traditional therapies against SHPT (VDRA and phosphate binders), was recently investigated by the EVOLVE trial [100]. As in the ACHIEVE and ADVANCE study, VDRA therapy was not controlled by the EVOLVE protocol, with the exception of VDRA adjustments in the case of iPTH < 150 pg/mL or hypocalcemia. Thus, the EVOLVE trial was not designed to assess a head to head VDRA and cinacalcet but rather the impact of cinacalcet on survival in addition to the more heterogeneous ongoing treatments of SHPT. At study end, a statistically nonsignificant trend toward reduction of the composite endpoint (time to death, myocardial infarction, hospitalization for unstable angina, heart failure, or a peripheral vascular event) was reported [relative hazard in the cinacalcet versus the placebo group 0.93 (95% CI: 0.85 to 1.02), *P* = 0.11)] [99]. However, the lower the anticipated event rate, the higher drop-in and -out rate during followup (about 20%) significantly affected the statistical power (0.54) and the interpretability of this inconclusive RCT [100]. 

Increasing the dose of cinacalcet or VDRA could be similarly effective on PTH suppression, with different effects on calcium-phosphate metabolism consequent to the VDR versus the calcium sensing receptor activation. Furthermore, the peculiar effects of each VDRA encourages further RCTs to test cinacalcet against a particular VDRA rather than a heterogeneous VDRA menu. Although the promising results of the ADVANCE and the EVOLVE trials, any superiority of calcimimetics versus vitamin D, or vice versa, on VC progression and hard end points is far from being established.

## 6. Conclusions

 An expanding body of evidence is rapidly enriching the rationale for vitamin D use in CKD-MBD. The traditional action of VDRAs on PTH suppression is now flanked by encouraging data on their pleiotropic effects on microalbuminuria and LVH. Furthermore, nutritional Vitamin D is receiving a growing interest as a preventive and treating strategy against SHPT as well as a protective intervention on immune responses, insulin resistance, and inflammation even in renal patients. However, further RCTs are advocated to investigates the many opened questions and uncertainties on the effects of VDRA and nutritional vitamin D on hard end points and their comparison with calcimimetic in CKD.

## Figures and Tables

**Table 1:** Vitamin D sterols currently available as medical treatments in nephrology field.

	Nutritional vitamin D		VDRA	
		Hydroxylation required to activate VDR		Hydroxylation required to activate VDR
Vitamin D2 and its analogs	Ergocalciferol	25-hydroxylation and 1-hydroxylation	Paricalcitol 19-nor1,25(OH)_2_D_3_	—
			Doxercalciferol 1a(OH)D_2_	25-hydroxylation

Vitamin D3 and its analogs			Calcitriol 1,25(OH)_2_D_3_	—
	Cholecalciferol	25-hydroxylation and 1-hydroxylation	Alfacalcidol 1a(OH)D_3_	25-hydroxylation
	Calcifediol	1-hydroxylation	Oxacalcitriol 22oxa1,25(OH)_2_D_2_	—

Note: all the VDRA reported in the table are considered analogs with the only exception of calcitriol, which corresponds to the natural form of 1,25(OH)_2_D_3_.

LVDRA: vitamin D receptor activators; VDR: vitamin D receptor.

**Table 2:** Burning questions on vitamin D prescription in CKD-MBD.

VDRA	Nutritional vitamin D	VDRA or calcimimetic
(i) Are VDRA superior to placebo in terms of cardiovascular events and survival? (ii) Do vitamin D analogs provide a better achievement of patient centered outcomes compared to calcitriol? (iii) Is any VDRA superior to the others in achieving KDIGO targets and improving albuminuria, LVH, VC, bone health, hospitalizations, and survival? (iv) Will paricalcitol ameliorate CKD progression and cardiovascular events through the benefits on albuminuria and LVH? (v) Should VDRA be suspended in those patients reaching PTH levels ≤ 150 pg/mL?	(i) Which are the optimal thresholds independently linked to SHPT and survival? (ii) Which is the best nutritional vitamin D regimen in terms of type and doses to replenish deficiency and treat SHPT? (iii) Will the replenishment be a cost-effective prevention against SHPT and CKD-MBD? (iv) Will the replenishment improve CKD progression, diabetes, infections, and survival? (v) Will the coadministration of native and active vitamin D be additive against CKD-MBD, infections, diabetes, and mortality?	(i) Is a VDRA-centered superior to a calcimimetic-centered therapy to control SHPT and survival? (ii) Which is the best cost-effective strategy in CKD-MBD: VDRA alone, calcimimetic alone, or a balanced association of VDRA and calcimimetic?

CKD: chronic kidney disease; SHPT: secondary hyperparathyroidism; LVH: left ventricular hypertrophy; VC: vascular calcification; VDRA: vitamin D receptor activators.
